# X-Ray Phase-Contrast CT of a Pancreatic Ductal Adenocarcinoma Mouse Model

**DOI:** 10.1371/journal.pone.0058439

**Published:** 2013-03-11

**Authors:** Arne Tapfer, Rickmer Braren, Martin Bech, Marian Willner, Irene Zanette, Timm Weitkamp, Marija Trajkovic-Arsic, Jens T. Siveke, Marcus Settles, Michaela Aichler, Axel Walch, Franz Pfeiffer

**Affiliations:** 1 Department of Physics and Institute of Medical Engineering, Technische Universität München, Garching, Germany; 2 Department of Radiology, Klinikum rechts der Isar, Technische Universität München, Munich, Germany; 3 Medical Radiation Physics, Clinical Sciences, Lund University, Lund, Sweden; 4 European Synchrotron Radiation Facility, Grenoble, France; 5 Synchrotron Soleil, Gif-sur-Yvette, France; 6 2nd Department of Internal Medicine, Klinikum rechts der Isar, Technische Universität München, Munich, Germany; 7 Research Unit Analytical Pathology, Institute of Pathology, Helmholtz Zentrum München, Oberschleißheim, Germany; University of Navarra, Spain

## Abstract

To explore the potential of grating-based x-ray phase-contrast computed tomography (CT) for preclinical research, a genetically engineered mouse model of pancreatic ductal adenocarcinoma (PDAC) was investigated. One ex-vivo mouse specimen was scanned with different grating-based phase-contrast CT imaging setups covering two different settings: i) high-resolution synchrotron radiation (SR) imaging and ii) dose-reduced imaging using either synchrotron radiation or a conventional x-ray tube source. These experimental settings were chosen to assess the potential of phase-contrast imaging for two different types of application: i) high-performance imaging for virtual microscopy applications and ii) biomedical imaging with increased soft-tissue contrast for in-vivo applications. For validation and as a reference, histological slicing and magnetic resonance imaging (MRI) were performed on the same mouse specimen. For each x-ray imaging setup, attenuation and phase-contrast images were compared visually with regard to contrast in general, and specifically concerning the recognizability of lesions and cancerous tissue. To quantitatively assess contrast, the contrast-to-noise ratios (CNR) of selected regions of interest (ROI) in the attenuation images and the phase images were analyzed and compared. It was found that both for virtual microscopy and for in-vivo applications, there is great potential for phase-contrast imaging: in the SR-based benchmarking data, fine details about tissue composition are accessible in the phase images and the visibility of solid tumor tissue under dose-reduced conditions is markedly superior in the phase images. The present study hence demonstrates improved diagnostic value with phase-contrast CT in a mouse model of a complex endogenous cancer, promoting the use and further development of grating-based phase-contrast CT for biomedical imaging applications.

## Introduction

X-ray computed tomography (CT) is a versatile 3D imaging technique. It features high spatial resolution, short acquisition time and is routinely used in clinical diagnosis and therapy response monitoring. Image contrast is generated by absorption and incoherent scattering processes of x-rays passing through the object of interest. This attenuation mechanism yields high contrast for strongly mineralized tissues, such as bones or teeth. However, only rather low contrast is achieved for soft tissues. This limitation can be overcome by the injection of a contrast agent (CA), resulting in soft-tissue contrast based on differences in CA uptake and washout dynamics.

A physically different effect can be explored with phase-sensitive x-ray imaging techniques. These rely on the phase shift that x-rays undergo when passing through matter [1]. The resultant refraction angle can be utilized as contrast mechanism in a grating interferometer in radiographic [2] and tomographic acquisition mode [3], [4]. This method provides two types of images at the same time: attenuation-contrast images and phase-contrast images. Grating interferometry can be used with both synchrotron radiation sources and conventional x-ray tube sources [5]. Recent studies in the last years in both configurations have shown excellent imaging results with respect to soft-tissue contrast [6]–[16]. Due to the compatibility of the method with x-ray tube sources, a translation to a clinical scenario is currently under discussion in the research community. Nevertheless, several challenges of mostly technological nature still have to be addressed. These relate in particular to the production of sufficiently large and sufficiently efficient gratings for typical acceleration voltages of about 120 kVp in human CT scanners with typical fields-of-view (FOV) of about 70 cm, or in mammography setups with FOVs of about 25 cm. Gratings of this size are as yet not available. However, already presently, small animal phase-contrast imaging is within reach, with lower mean x-ray energies, a smaller FOV and relaxed dose restrictions in comparison to human applications. Development efforts of a first small animal grating-based phase-contrast CT scanner are pursued and first experimental results have been shown [17], [18].

This study focuses on the assessment of x-ray phase-contrast imaging for preclinical research. In this context, the potential of the technique for two different possible applications is investigated. On the one hand we study high-performance, high-dose, and high spatial resolution imaging for virtual microscopy applications such as high-throughput therapeutic response monitoring, anatomical phenotyping or as tool for pre-histological investigations. This section is covered by the performed SR-based benchmarking experiment. On the other hand the capability of phase-contrast CT for in-vivo imaging with improved soft-tissue contrast is assessed with dose-reduced measurements of the same mouse specimen using synchrotron radiation and using a conventional tube source. The two latter settings represent idealized preclinical imaging systems with the limitation that the specimen, rather than the CT gantry, was rotated. Moreover, magnetic resonance imaging was performed on a clinical 1.5 Tesla MRI scanner using a dedicated microscopy coil. The MRI measurement serves as reference technique with well-established high intrinsic soft-tissue contrast due to differing T1 recovery and T2 decay times. Finally, as the gold standard technique for tissue classification, histology was obtained for a limited section of the mouse specimen. The potential of high-performance phase-contrast CT for virtual microscopy applications and of dose-reduced phase-contrast CT for in-vivo imaging was investigated based on a comparison of attenuation and phase images. For both cases, this comparison is done qualitatively and quantitatively using contrast-to-noise ratios.

## Results and Discussion

### X-ray dose considerations

To assess dose compatibility with respect to future in-vivo imaging applications, the air dose was determined. It should be noted, however, that the setups were not specifically optimized for dose-this relates in particular to the imaging detectors, which featured a low detective quantum efficiency (DQE). For this reason, two dose values were determined: the actually delivered dose and a feasible dose value. The latter is calculated from the measured dose and is based on a higher DQE, corresponding to existing efficiency-optimized detectors, and thinner grating support (within the limits of technical feasibility), reducing the loss of x-rays due to attenuation. Details about the measurement of x-ray dose and derivation of the feasible dose value can be found in a dedicated subsection of the Materials and Methods section. Table 1 lists the determined actual and feasible dose values. As expected, the feasible dose value of 40 Gy for the high-performance synchrotron measurement is substantial and by far not compatible with in-vivo imaging. This is different for the low-dose measurement with a feasible dose value of 0.4 Gy. Similarly, for the tube source measurement, where the feasible dose value amounts to 1.1 Gy. For comparison, dose values of up to several hundred mGy for in-vivo microCT imaging of mice are reported in the literature[19]. Regarding the CT measurements performed for our studies, there is room for further dose reduction even beyond the feasible dose scenario. For example Zanette et al. recently reported a data acquisition and processing method that optimizes dose efficiency by a factor of 4, while maintaining image quality [20]. This means that-assuming a dose-optimized setup-the low-dose synchrotron and tube measurement would reach a range that is compatible with in-vivo imaging of small animals. In the light of these considerations, both dose-reduced measurements hence probe the maximum achievable image quality for phase-contrast CT in-vivo imaging applications.

**Table 1:** Determination of x-ray air dose.

	Actual dose [Gy]	Feasible dose [Gy]
**Synchrotron**		
high-performance	430 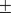 110	40 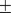 10
low-dose	4.3 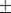 1.1	0.4 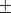 0.1
**Tube source**	6.9 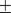 1.7	1.1 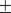 0.3

The actually measured air dose and a feasible dose value for an optimized experimental setup are listed.

### Overview of imaging data and analysis

The abdomen of the ex-vivo mouse specimen with induced PDAC was imaged with the mentioned x-ray phase-contrast setups and MRI. Subsequently, histology images of a limited section of the mouse specimen were acquired. All synchrotron measurements were performed using a two-grating Talbot interferometer at the European Synchrotron Radiation Facility (ESRF, Grenoble, France). For the high-performance measurement the focus was on optimizing image quality disregarding x-ray dose, as opposed to the dose-reduced measurement with the focus on minimizing dose in a synchrotron setup. The second dose-reduced measurement was performed using a three-grating Talbot-Lau interferometer and conventional x-ray tube source. The x-ray energy of 35 keV for the synchrotron setup was chosen to optimize image quality. For the three-grating tube setup, the acceleration voltage was chosen to optimize visibility and was set to 35 kV. This acceleration voltage results in an effective energy of 23 keV, which is the design energy of the interferometer. Further details about the preparation of the mouse specimen and the technical parameters of the imaging setup can be found in the Materials and Methods section.

**Figure 1:**
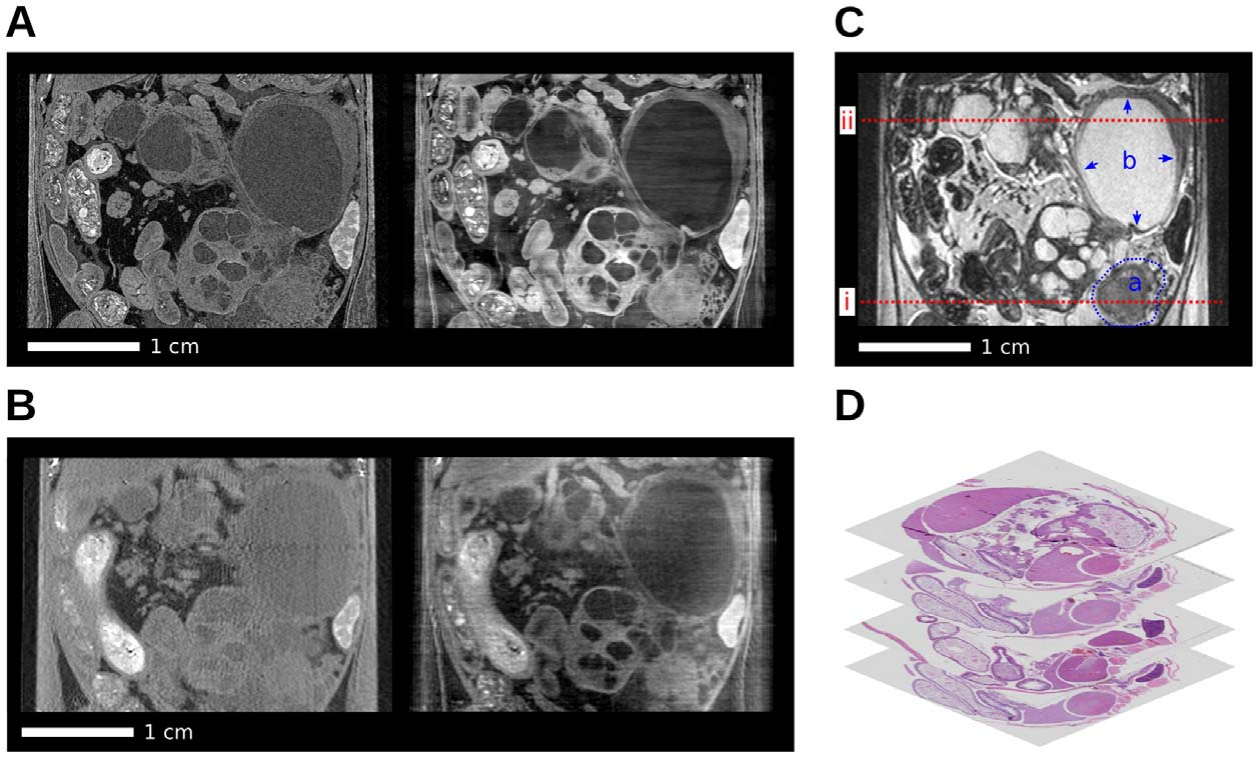
Coronal slices of the acquired multi-modal tomographic imaging data of the abdominal area in the mouse and histology. (A) Synchrotron: Attenuation-contrast image (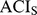) (left), phase-contrast image (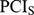) (right). (B) Tube source: Attenuation-contrast image (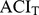) (left), phase-contrast image (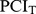) (right). (C) MRI with highlighted solid tumor (a) and cystic lesion (b). (D) Stack of histology slices. All images are displayed on a linear gray scale and are windowed for best visual appearance of the solid tumor and cystic lesion.

Fig. 1 gives an overview over the imaging data (except for the synchrotron low-dose measurement) of the abdominal area of the mouse. It shows coronal x-ray and MR images and exemplary axial histology slices. The high-performance synchrotron measurement with attenuation (left) and phase contrast (right) is shown in panel (A). Panel (B) shows the corresponding tube source measurement, (C) the T2-weighted (T2w) MRI data and (D) histology slices. In order to distinguish between the different x-ray imaging setups and image contrasts, the following notation will be used: ACI-attenuation contrast image, PCI-phase contrast image, index S for synchrotron radiation source and index T for tube source. Due to the differences in imaging parameters, there is a difference in effective pixel size between the high-performance synchrotron (30 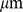), the tube-based (120 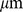) and the MRI data (130 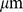). Bone as a strongly phase-shifting tissue causes streaking artifacts in transverse phase slices, similar to metal artifacts in conventional CT. These streaking artifacts appear as horizontally oriented intensity fluctuations in the shown coronal phase images. The pancreatic tumor induction gave rise to the formation of solid tumor tissue and various lesions as previously described [21], [22]. These included pancreatic intraepithelial neoplasia (PanIN) lesions progressing to invasive PDAC as well as dilated, cyst-like ducts probably due to regional obstruction. In the MRI reference image (panel C), this solid tumor tissue (a) and one large cystic lesion (b) are highlighted. The further analysis of the image data is based on transverse slices at the indicated positions (red dashed lines) and is divided into two parts. To examine the potential of x-ray phase-contrast CT for in-vivo applications, the visibility of the solid tumor tissue (a), under dose-reduced conditions, is considered. The potential for virtual microscopy applications on the other hand is based on the high-performance data of the indicated cystic lesion (b). For both cases, x-ray attenuation and phase images from one experimental setup are compared with one another. This comparison is done both visually and quantitatively using contrast-to-noise-ratios of selected regions-of-interest. Please note that this comparison is only performed for attenuation and phase images from the same imaging setting and not across settings as the experimental parameters and especially the spatial resolution are different.

### Potential of phase-contrast CT for in-vivo imaging

**Figure 2:**
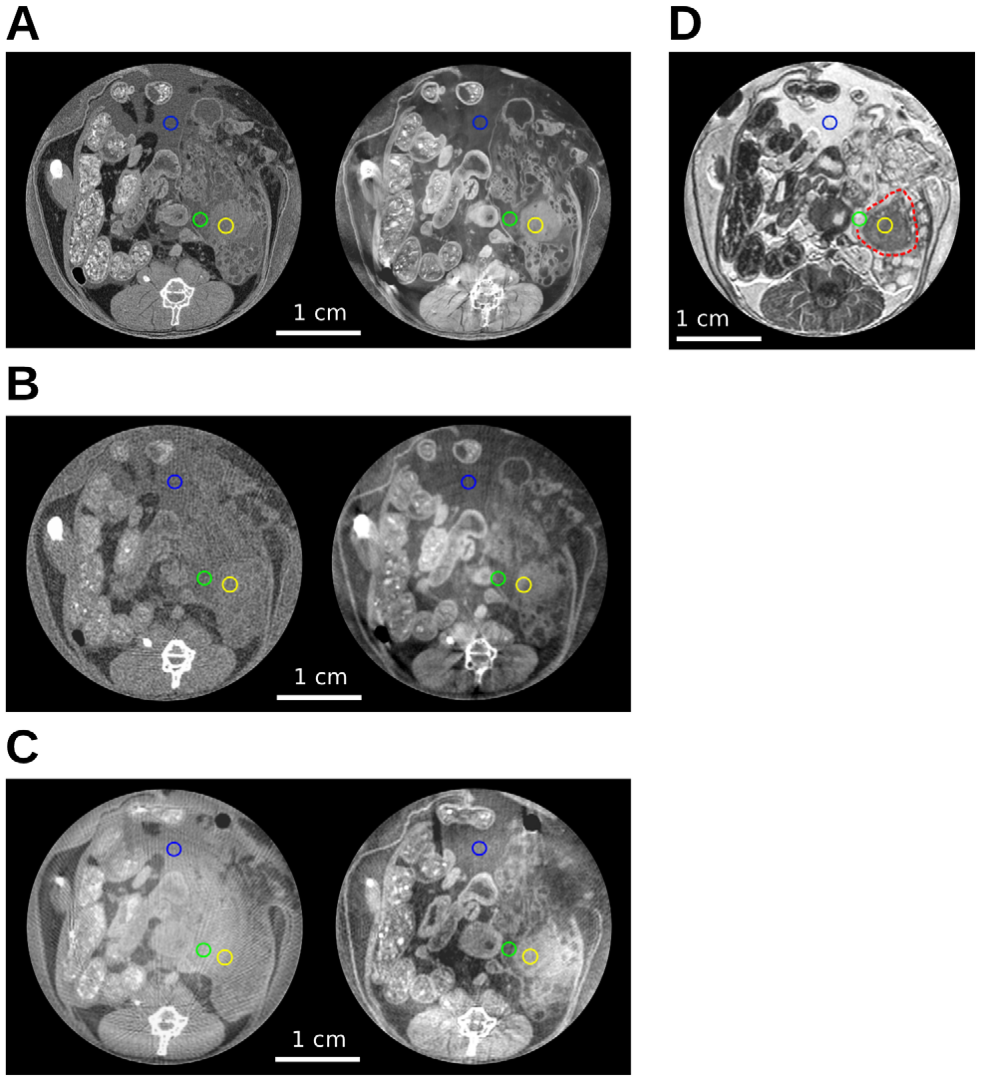
Assessment of solid tumor visibility. (A) Synchrotron: 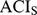 (left), 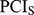 (right). (B) Synchrotron (low dose): 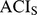 (left), 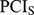 (right). (C) Tube source: 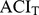 (left), 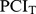 (right). (D) MRI, solid tumor part indicated by red dashed line. Regions-of-interest for the quantitative contrast-to-noise ratio analysis are indicated by colored circles. All images are displayed on a linear gray scale and are windowed for best visual appearance of the solid tumor.

As phase-contrast CT increases soft-tissue contrast, potential applications for preclinical imaging include non-invasive tumor detection and characterization. In the present mouse specimen, the solid tumor tissue is regarded for this purpose. Fig. 2 shows transverse slices (position indicated by profile 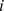 in Fig. 1) for all three x-ray imaging settings [(A)–(C)] with attenuation contrast on the left and phase contrast on the right side. As a reference, the MRI image is shown in (D). Panel (A) contains the high-performance and (B) the low-dose SR-based data. Panel (C) depicts the tube-based images. The high-performance SR-based data is shown for completeness, but naturally the dose-reduced imaging data (B and C) is of most interest for evaluating the potential for in-vivo imaging. Tumor detection by means of imaging is mainly based on a detailed knowledge of anatomy and the morphology of abnormal tissue. For the present mouse specimen, solid tumor tissue was identified at the indicated position in the MRI reference image (D). In the high-performance SR-based data (A), this tissue can be identified clearly in both images, however recognizability is superior in the phase image. Visibility of this tissue is also given in the phase image of the low-dose SR-based data (B), but is very poor in the corresponding attenuation image. The same applies to the tube measurement (C)-also here, identification of the solid tumor mass is clearly possible on the basis of the phase image, but is strongly compromised in the attenuation image. This means that, for the imaging settings that assess preclinical in-vivo imaging applications, tumor visibility is strongly superior in the phase images. This observation demonstrates the potential of phase-contrast CT for tumor detection.

In order to objectify and quantify this evaluation of tumor visibility, a CNR analysis of selected ROIs was performed. Three ROIs were selected for each image: 1) covering the solid tumor, 2) covering surrounding tissue, 3) probing image noise. A separate ROI for determining noise was used because the standard deviation in a ROI reflects two opposing effects: image noise and tissue heterogeneity. Image noise should of course be considered in the CNR, unlike tissue heterogeneity that, if present within the ROI, will also increase the standard deviation and hence falsely appear as additional noise. The third ROI is hence placed within a homogenous region, and the standard deviation only reflects image noise. The CNR, based on the mean values of region 1 and 2 (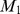 and 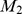) and the standard deviation of region 3 (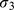), is then calculated according to:
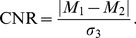
(1)


The size of the ROIs was chosen to cover an area of 1 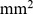, which is as large as reasonably possible to only cover the specific tissue of interest. In Fig. 2, the chosen ROIs are displayed in each image as colored circles: yellow covering solid tumor tissue (1), green covering surrounding tissue (2), and blue covering a homogeneous region (3). Table 2 lists the individual CNRs as well as their respective ratio that reflects the relative contrast improvement of phase over attenuation contrast, i.e. the relative contrast gain. The uncertainty of the CNR (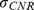) was determined by applying standard error propagation to the equation of the CNR (equation 1). The required uncertainties are the standard error (SE) of each mean value (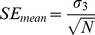) and the standard error of the noise estimate (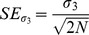). 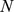 denotes the number of voxels in the corresponding ROI [23]. Subsequently, error propagation was also applied to the relative contrast gain values, based on the determined uncertainties of each CNR (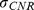). The uncertainties for all CNRs and relative contrast gain values are quoted as errors in Table 2. The relative contrast gain values range from 4 for the low-dose SR-based images, over 10 for the tube images, up to 28 for the high-performance SR-based images. The tube data are obviously of particular interest for in-vivo applications. The contrast improvement of 10 for the phase images from the tube setup compared to the attenuation images clearly underlines the strong potential of phase-contrast CT for early tumor detection for in-vivo applications. As a reference, the absolute CNR of the magnetic resonance images amounts to 8.0 (not listed in Table 2). Please note that a comparison of CNRs across different imaging setups is not directly meaningful due to the difference in spatial resolution.

**Table 2:** Contrast-to-noise ratio analysis for solid tumor visibility and tissue composition discernibility.

	Attenuation contrast	Phase contrast	Relative contrast gain
**Solid Tumor**			
Synchrotron (high-performance)	0.37 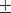 0.04	10.4 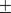 0.2	28 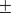 3
Synchrotron (low-dose)	0.6 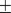 0.2	2.4 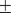 0.3	4 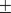 1
Tube source	0.8 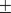 0.2	7.8 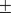 0.6	10 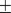 3
**Cystic lesion**			
Synchrotron (high-performance)	0.06 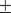 0.08	7.2 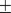 0.3	—

**Figure 3:**
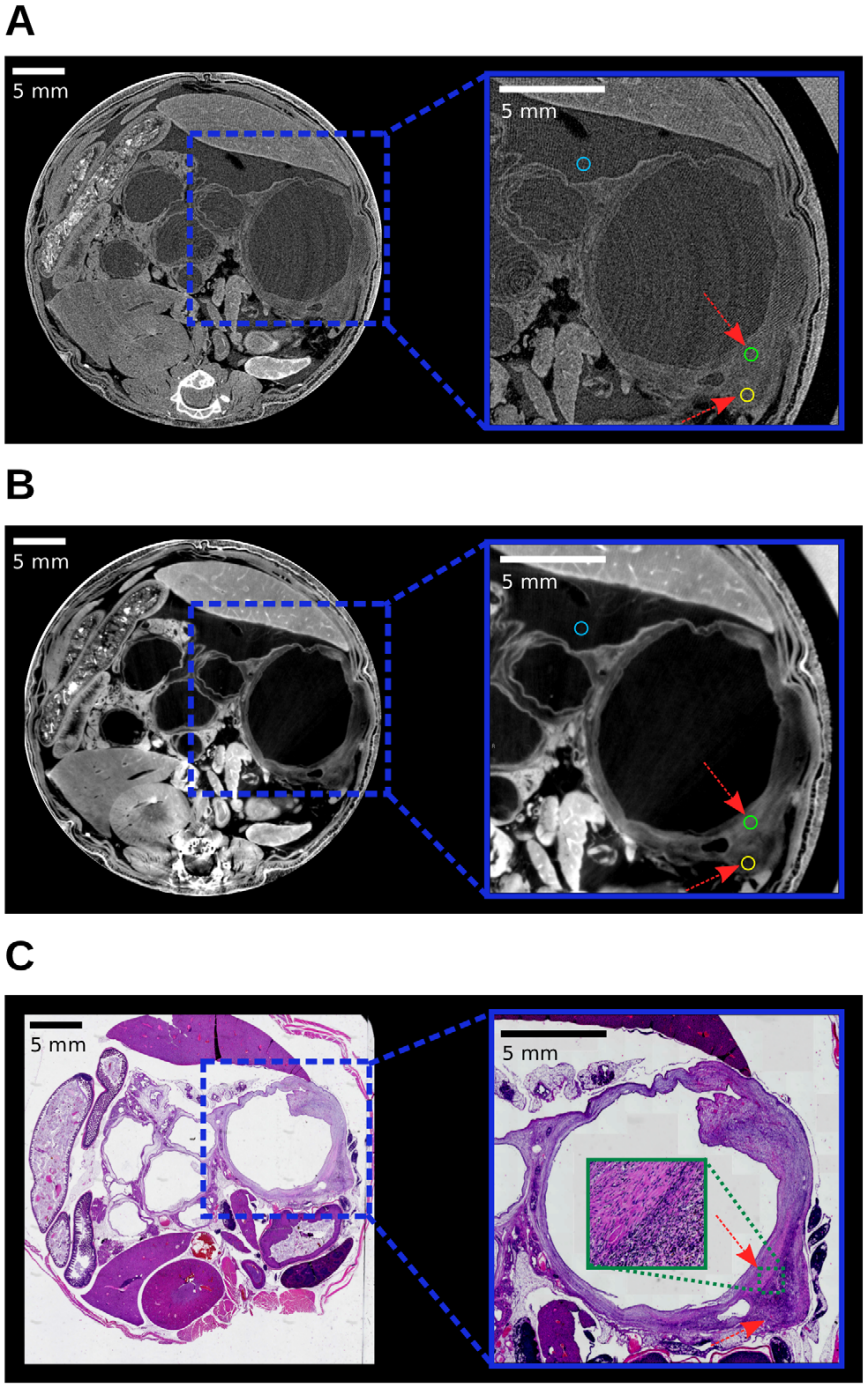
Assessment of tissue composition discernibility. (A) 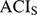 (left), Zoom (right). (B) 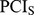 (left), Zoom (right). The arrows highlight positions of differences in tissue composition. (C) Histology (left), Zoom (right). Regions-of-interest for the quantitative contrast-to-noise ratio analysis in the x-ray images are indicated by colored circles. All images are displayed on a linear gray scale and are windowed for best visual appearance of the cystic lesion.

Based on the indicated ROIs in Fig. 2 and Fig. 3, the CNRs for attenuation and phase contrast, as well as their ratio (relative contrast gain), are listed. For the cystic lesion, the relative contrast gain is not listed as the error in the attenuation image is of the same order as the CNR itself.

### Potential of phase-contrast CT for virtual microscopy applications

Increased soft-tissue contrast in combination with the capability of high spatial resolution renders phase-contrast CT attractive for virtual microscopy applications. This potential area of application was examined on the basis of the high-performance SR-based CT data of the previously indicated cystic lesion. In tumor biology in general, and specifically for tumor characterization and therapy response monitoring, tissue heterogeneity is recognized as a characteristic feature. This is, for example, because differences in tissue composition result in regional differences in therapy response. Such a difference in tissue composition is for example present around the mentioned cystic lesion and is examined in detail in the following. Fig. 3 shows transverse slices of the SR-based high-performance attenuation (A) and phase-contrast data (B) and the corresponding histology slice (C). It should be noted that the orientation of the x-ray and histology slice are marginally tilted with respect to one another, with best agreement at the lower right part of the cystic lesion, which is investigated. Moreover, in the histologic slice the upper right part of the wall of the lesion is locally deformed due to the cutting procedure. Moreover, strain fields in histological slices can lead to further deformations of the original shape [24]. Most relevant for this study is the well-known tissue shrinkage caused by dehydration. While the misalignment and deformation were small enough to be tolerable for the analysis in the present case, suitable registration algorithms exist that can be used to align the tomography images with the 2D histology data and remove distortions in the general case [25]. The mentioned difference in tissue composition, either fibrotic or cell-rich stroma, can be observed in the zoomed area of the histologic slice (C). The arrows indicate these two areas and the border in between is displayed in the extra zoom panel. This difference in tissue composition is also accessible in the phase image (B), i.e. at the indicated positions the cell-rich stroma appears darker and the fibrotic part brighter. In the attenuation image this information is not accessible and the surrounding tissue of the cyst appears homogeneous.

This visual impression was also analyzed quantitatively as before on the basis of the three indicated ROIs in Fig. 3 (area of 0.3 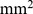). The corresponding CNRs and associated uncertainties are also listed in Table 2. The very low CNR of 0.06 in the attenuation image, and the significant CNR of 7.2 in the phase image reflect the described visual impression that the discrimination of tissue composition is practically impossible in the attenuation image, but is well represented in the phase images. The relative contrast gain was not determined for this set of images as, in the attenuation image, the error in the CNR of 0.08 is on the same order as the CNR itself. This demonstrated capability of phase-contrast CT of visualizing such subtle differences in tissue composition underlines the potential of phase-contrast CT for virtual microscopy applications.

### General Discussion

For the assessment of the potential of phase-contrast CT for preclinical imaging, we investigated different x-ray phase-contrast imaging settings. As very well-established reference technique with high intrinsic soft-tissue contrast, MRI was used. In this regard, we would like to indicate a technical limitation of our study. The employed instrumentation of a human MR scanner with microscopy coil is not ideally suited for microscopic imaging and dedicated small animal MR scanners are available in principle. These scanners are optimized for smaller samples and perform superior with respect to spatial resolution and contrast. For our purposes however-to include a well-established soft-tissue imaging technique as a reference-the performance of the MR scanner was sufficient. For an elaborate comparison of phase-contrast CT and MRI, the reader is referred to a recent study by Schulz et al. [26], comparing phase-contrast CT, magnetic resonance microscopy and histology of the human cerebellum.

Concerning the signal-to-noise ratio of attenuation and phase contrast in general, it has been reported in the literature that phase contrast benefits from comparably small detector pixel sizes [27]–[29]. In preclinical imaging systems, pixel sizes naturally are smaller than in human CT scanners, rendering phase contrast especially attractive for preclinical imaging applications.

When comparing attenuation and phase-contrast images from a grating-based setup in general, one aspect relating to the method should be noted. In a conventional CT setup, there are no gratings present and the dose-relevant final grating (G2) absorbs approximately half of the x-rays that would otherwise also contribute to the image signal. This means that attenuation-contrast images from a grating-based setup require approximately twice the dose needed for comparable images from a conventional setup. On the other hand, the grating-based setup yields-from a single CT scan with a certain radiation dose-two perfectly registered images with complementary contrast mechanisms.

## Conclusion

The potential of grating-based phase-contrast CT for preclinical imaging applications was investigated on the basis of multi-modal image data of an ex-vivo pancreatic ductal adenocarcinoma mouse model specimen. Besides reference imaging using an MR scanner and tissue classification on the basis of histology, two different x-ray phase-contrast settings were used in the study: on the one hand dose-reduced imaging using synchrotron radiation, and using a conventional x-ray tube source and on the other hand synchrotron radiation imaging for benchmarking. To assess the potential of phase-contrast CT for small animal in-vivo imaging, the visibility of solid tumor tissue was compared in attenuation and phase images for both dose-reduced datasets. It was found both visually and quantitatively by means of CNRs that the increased soft tissue contrast apparent in phase images does allow for tumor identification, unlike in the attenuation images. The evaluation of phase-contrast CT for virtual microscopy applications was based on the visibility of subtle differences in tissue composition of a cystic lesion in the high-performance SR-based data. Here, it was observed visually and quantitatively on the basis of CNRs that the phase images do display these differences in tissue composition, as opposed to the attenuation images. The study hence demonstrates the potential of phase-contrast CT exemplary for both types of preclinical imaging applications and we believe that this study facilitates the use and further development of this technique.

## Materials and Methods

### Ex-vivo mouse specimen

A mouse with pancreas specific activation of oncogenic Kras was obtained by breeding 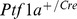 knock-in mice with 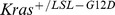 animals [21]. To model PDAC, a 19 months old 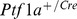 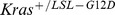 (CK) animal was used. A T2-weighted anatomy MRI scan of CK mice of tumor-bearing age was performed to confirm the presence of a solid tumor mass. Subsequently the animals were kept in isoflurane narcosis and a median laparotomy was performed, followed by a perfusion fixation protocol. In brief, the left ventricle was canulated with a 22 G needle, followed by clipping of the right atrium. Then, 10 ml of phosphate buffered saline was manually infused to flush out all blood from the vasculature, followed by manual infusion of 20 ml 4% paraformaldehyde (PFA). Thereafter the animal was emerged for 72 hours in 200 ml 4% PFA, briefly washed in 70% ethanol and transferred into a 50 ml Falcon tube in 70% ethanol. Animal care and experimental protocols were conducted in accordance with German animal protection laws and approved by the Institutional Animal Care and Use Committee at the Technische Universität München. For histology, the mouse was removed from the plastic container. The abdomen was cut off, dehydrated in an automated tissue processor and embedded in paraffin. After embedding, the spine was removed and the whole abdomen was cut into 4 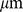 thick serial sections. Afterwards, the sections were stained with hematoxylin and eosin and digitalized with a slide scanner (DotSlide, Olympus).

### X-ray grating-based phase-contrast CT

Conventional x-ray attenuation-contrast images and phase-contrast images were obtained using a grating interferometer [2], [4], [5]. The synchrotron radiation source measurements were carried out at beamline ID 19 of the European Synchrotron Radiation Facility (ESRF) in Grenoble, France. Images were recorded with a two-grating Talbot interferometer and scintillator/lens-coupled CCD detector (FReLoN) [30]. The interferometer was operated with a monochromatic x-ray beam of 35 keV (Si(111) double-crystal monochromator, energy bandwidth 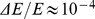). The exact interferometer and tomography parameters are listed in Table 3 and Table 4 respectively. For the low-dose dataset, the detector pixels were binned 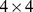 prior to readout, resulting in an effective pixel size of 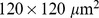. The tube source measurement was performed at the Physics Department of the Technische Universität München, Munich, Germany. A rotating anode (Enraf Nonius FR 591, molybdenum target) with an effective focal spot size of 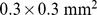 was operated at 35 kVp and 70 mA. With the three gratings in the beam (each on a silicon wafer of 500 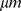 thickness acting as a filter), this results in an x-ray spectrum with its center at the design energy of 23 keV of the interferometer. Phase- and attenuation-contrast images were obtained with a three-grating Talbot-Lau interferometer and a Pilatus II silicon-based photon-counting imaging detector. For detailed parameters, see Table 3 and Table 4.

**Table 3:** Interferometer characteristics.

	Energy	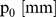	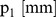	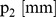	Eff. pixel size 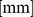	d [mm]	Talbot order
Synchrotron	35 keV	—	4.79	2.40	30/120	408	5
Tube source	23 keV	10.0	3.51	5.40	120	527	3

The x-ray energy, grating periods, effective pixel size, inter-grating distance d (between G1 and G2) and corresponding fractional Talbot order are listed. The quoted x-ray energy of 23 keV for the tube source specifies the center of the polychromatic spectrum.

**Table 4:** X-ray CT data acquisition parameters.

	# Projections	# Phase steps	Exposure time [s]
**Synchrotron**			
high-performance	901	4	1
low-dose	301	3	0.04
**Tube source**	301	10	12

The data acquisition parameters of the CT scans for the different imaging settings are listed.

### Measurement of x-ray dose

For determining the x-ray dose in each measurement, a clinically approved dosimeter (Patient Skin Dosimeter, Unfors, Sweden) was used. This dosimeter is designed for monitoring the x-ray dose during fluoroscopic CT procedures and is calibrated for entrance skin dose at 90 kVp. When taking a conversion factor of 1.4 into account, the dose in free air can be determined from the displayed entrance skin dose (Unfors PSD, Patient Skin Dosimeter, User's Manual). As the difference in effective energy for the various experimental setups was not considered, the determined dose value is subject to some uncertainty. However, a precise dosimetry of the performed CT measurements was not the objective of the study and the dose value rather serves the purpose of evaluating the compatibility with preclinical in-vivo imaging. In the technical specifications, the manufacturer quotes an energy dependence of 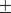 15% (40 kVp–150 kVp) and an uncertainty of 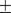 6%. The error in the determined dose values, for good measure, is hence assumed to be within 25%. As both the synchrotron and tube source imaging setup are not optimized for dose, two dose values were determined: the actually delivered dose and a feasible dose value. The latter value is calculated from the experimentally measured one and is based on two premises: 70% detective quantum efficiency (DQE) and a thinner silicon wafer support of the gratings. A DQE of 70% is used since, for example state-of-the art CMOS imaging detectors reach such high DQE at small pixel sizes of 75 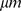 (Dexela 2923) [31]. The calculation of feasible dose is based on the actual DQE of the used detector and a subsequent scaling to 70%. Thinner silicon wafers are based on a thickness of 100 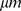, instead of the actual thickness of 500 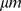. The scaling effect in dose due to reduced attenuation in the thinner wafer is calculated mono-energetically and is based on the present synchrotron photon energy of 35 keV and 23 keV interferometer design energy for the tube setup. For the synchrotron setup, the CCD detector (FReLoN) sensitivity was determined from the DQE at zero spatial frequency 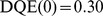, from Coan et al. [32] (Table 3, first column, 33 keV). The scintillator screen thickness of the FReLoN detector in our study was 30 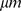, as opposed to a thickness of 100 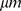 in Coan et al. [32]. In order to account for the thinner scintillator screen-and related lower DQE-in our study, the reduced attenuation of x-rays in the thinner scintillator screen was considered and the DQE value of 0.30 was scaled accordingly. This scaling results in a DQE of 0.11 for our measurement. The thinner silicon wafers were taken into account by considering the reduced attenuation in both gratings of the synchrotron setup. For the tube source setup, the DQE was determined from the attenuation of 23 keV photons (interferometer design energy) in the sensitive silicon layer of 450 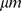 in the Pilatus II detector and amounts to 0.25. Thinner silicon wafers were again considered by the reduced attenuation in all three gratings. Both the actual and feasible dose value for the different imaging setups are quoted in Table 1.

### Magnetic resonance imaging

High resolution MR imaging was performed on a 1.5 T clinical scanner (Philips Achieva) using the 47 mm diameter microscopy coil. The sequence was a 3D turbo spin echo (TSE) sequence with an isotropic resolution of 130 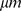 and the following parameters: FOV = 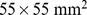, TR = 1000 ms, TE = 95 ms, turbo factor 15, echo train length 333 ms, 6 NSA and a total acquisition time of approximately 14 hrs. A DRIVE pulse was used to compensate for the short repetition time.

### CT Reconstruction, image processing and data analysis

CT reconstruction was performed using a standard filtered backprojection algorithm. For attenuation contrast, a Ram-Lak filter and for phase contrast, a Hilbert filter was used [33]. Strongly phase shifting materials, such as bone, produce streaking artifacts. To reduce the magnitude of the streaks, a straightforward method was applied during reconstruction of the phase-contrast data, i.e. the differential phase sinogram was weighted with the squared value of the interferometer visibility. This approach suppresses the bone signal as bone scatters strongly and hence causes a low weighting of the corresponding area in the differential phase sinogram. This method however only reduces the magnitude and streaking artifacts are still present. All attenuation- and phase-contrast CT slices were post-processed with a sharpening filter. Minor deformations in the shape of the mouse and changes in the position of few air bubbles are apparent between the different measurements and image registration was performed manually. Please note that the attenuation and phase image of each tomography however are intrinsically perfectly registered with one another as both images originate from one common dataset. Each image is displayed on a linear gray scale and windowed for best visual appearance of the discussed features. Comparison of image contrast is then done visually and quantitatively using CNRs.
